# Multipotent luminal mammary cancer stem cells model tumor heterogeneity

**DOI:** 10.1186/s13058-015-0615-y

**Published:** 2015-10-14

**Authors:** Lei Bao, Robert D. Cardiff, Paul Steinbach, Karen S. Messer, Lesley G. Ellies

**Affiliations:** Bioinformatics and Biostatistics Shared Resource, Moores UCSD Cancer Center, University of California San Diego, 3855 Health Sciences Drive, La Jolla, CA 92093 USA; Center for Comparative Medicine, University of California, 1 Shield Avenue, Davis, CA 95616 USA; Department of Pharmacology, University of California San Diego, 9500 Gilman Drive, La Jolla, CA 92093 USA; Department of Pathology, University of California San Diego, 9500 Gilman Drive, La Jolla, CA 92093-0612 USA; Divison of Biostatistics and Bioinformatics, Department of Family and Preventive Medicine, University of California San Diego, 9500 Gilman Drive, La Jolla, CA 92093 USA

## Abstract

**Introduction:**

The diversity of human breast cancer subtypes has led to the hypothesis that breast cancer is actually a number of different diseases arising from cells at various stages of differentiation. The elusive nature of the cell(s) of origin thus hampers approaches to eradicate the disease.

**Methods:**

Clonal cell lines were isolated from primary transgenic polyomavirus middle T (PyVmT) luminal tumors. Mammary cancer stem cell (MaCSC) properties were examined by immunofluorescence, flow cytometry, differentiation assays and *in vivo* tumorigenesis.

**Results:**

Clonal cell lines isolated from primary PyVmT mouse mammary luminal tumors can differentiate into luminal, myoepithelial, alveolar and adipocyte lineages. Upon orthotopic injection, progeny of a single cell follow a pattern of progression from ductal carcinoma *in situ*, to adenoma, adenocarcinoma and epithelial metastasis that recapitulates the transgenic model. Tumors can evolve *in vivo* from hormone receptor-positive to hormone receptor-negative Her2-positive, or triple negative CD44hi basal-like and claudin-low tumors. Contrary to the current paradigm, we have defined a model in which multiple tumor subtypes can originate from a single multipotent cancer stem cell that undergoes genetic and/or epigenetic evolution during tumor progression. As in human tumors, the more aggressive tumor subtypes express nuclear p53. Tumor cell lines can also be derived from these more advanced tumor subtypes.

**Conclusions:**

Since the majority of human tumors are of the luminal subtype, understanding the cell of origin of these tumors and how they relate to other tumor subtypes will impact cancer therapy. Analysis of clonal cell lines derived from different tumor subtypes suggests a developmental hierarchy of MaCSCs, which may provide insights into the progression of human breast cancer.

**Electronic supplementary material:**

The online version of this article (doi:10.1186/s13058-015-0615-y) contains supplementary material, which is available to authorized users.

## Introduction

Approaches to eradicate breast cancer have been hampered by the elusive nature of the cell(s) of origin that can give rise to a diverse group of tumors, some of which have the ability to metastasize. While current evidence suggests that hereditary breast cancer promoted by loss of Brca1 arises in luminal progenitor cells [1], the cell of origin for the majority of breast tumors, which are spontaneous in nature, remains undetermined. While putative breast cancer stem cells (CSCs) have been identified from a small subset of aggressive tumors [2], understanding whether the majority of breast cancers arise from CSCs, from clonal evolution of differentiated cells [3] or a combination of these mechanisms will impact strategies for cancer therapy.

Evidence suggests that human breast cancer originates in the terminal ductal lobular unit (TDLU), a monoclonal branching ductal-alveolar structure consisting of luminal and myoepithelial cells that is associated with limited ductal elongation in its normal microenvironment [4, 5]. Luminal tumors account for the majority (60 to 70 %) of human breast cancers [6, 7], but whether the cell of origin is a multipotent tissue stem cell, a committed luminal progenitor cell or a fully differentiated luminal cell is currently unknown. Furthermore, the relationship between normal mammary stem cells and breast CSCs is in a state of flux due in part to breast cancer heterogeneity, variations in marker analyses between different laboratories and the lack of cell-based model systems that faithfully recapitulate spontaneous luminal tumorigenesis and metastasis.

To investigate whether luminal tumors harbor CSCs, multiple clonal cell lines were derived from the transgenic polyomavirus middle T (PyVmT) oncogene mouse model of luminal tumorigenesis in both the C57Bl/6 and FVB/N strains [8, 9], by culture of enzymatically digested spontaneous tumors followed by limiting dilution cloning. We have focused this report on the C57Bl/6 Py230 cell line [10] because of its genetic stability, but similar phenotypes have been found with additional cell lines.

## Methods

### Generation and culture of mammary cancer stem cell lines

Spontaneous tumors from Tg(MMTV:LTR-PyVmT) mice congenic in the C57Bl/6 (B6.FVB-Tg(MMTV-PyVT)634Mul/LellJ) [9] or FVB/N (FVB-Tg(MMTV-PyVT)634Mul) [8] background were enzymatically digested in collagenase buffer: 1 mg/ml collagenase (type 2, Worthington Biochemical Corp., Lakewood, NJ, USA), 2 mg/ml soybean trypsin inhibitor (Sigma-Aldrich, San Louis, MO, USA), 1 mg/ml BSA (Sigma-Aldrich), 50 μg/ml gentamicin (Life technologies, Grand Island, NY, USA) in F12K media (Mediatech Inc., Manassas, VA, USA) for two to three hours at 37 °C, with shaking. The cloudy cell suspension was neutralized with serum containing media, filtered through a 70-μm mesh, spun down and resuspended in complete F12K media containing 5 % fetal clone II (Hyclone, Logan, UT, USA), MITO (1:1,000 dilution, BD Biosciences, San Jose, CA, USA), 50 μg/ml gentamicin and 2.5 μg/ml amphotericin B. The cells were maintained at high density for approximately five passages and then cloned by limiting dilution. Clonal cell lines were maintained in complete F12K media.

For cloning to measure self-renewal, clonal cell lines were cultured in ultra-low adhesion plates (Corning, Corning, NY, USA) in complete F12K media for 48 hours, during which time the majority of differentiated cells underwent apoptosis. The surviving cells were briefly trypsinized to separate small clumps of cells, and single cells were hand-picked and plated into the wells of a 96-well plate. After 10 to 14 days, the number of colonies per plate that were >20 cells per well was counted. Cells from four to six of these colonies were pooled to use for the next round of cloning. Four colonies per plate were individually expanded and injected into mice to confirm their tumor forming ability. Standard procedures were used to generate mammospheres [11], which were plated onto rat-tail collagen gels (type 1, Sigma-Aldrich) and cultured in complete media for up to 30 days. Confluent two-dimensional cultures spontaneously formed domes and were treated with 100 μM dexamethasone (Sigma-Aldrich) and 10 U/ml prolactin (Sigma-Aldrich) for 48 hours to stimulate ß casein expression. In other experiments, Py230 cells were plated at low density and treated with differentiating agent dimethyl sulfoxide (DMSO, Sigma-Aldrich) for 7 to 10 days, when outgrowth of mesenchymal cells could be observed. 10^6^ cells from the total cell population were injected *in vivo* and cells were cloned from the resulting tumor. The Py15-4 cell line generated from the Py230 luminal progenitor cell line was then characterized by array analysis and real-time PCR, as described below.

Growth curves were generated by plating 10^4^ cells per well into 24-well plates. Each day, triplicate cultures were counted using a Z series Coulter Counter (Beckman Coulter, Brea, CA, USA). To prepare chromosome spreads, cells were treated with 5 μg/ml nocodazole (Sigma-Aldrich) for six hours at 37 °C, harvested and resuspended in 0.56 % KCl (Sigma-Aldrich) hypotonic solution for 10 minutes. The cells were fixed in Carnoy’s solution (methanol:acetic acid, Sigma-Aldrich at 3:1), and the cell suspension dropped onto microscope slides (Thermo Scientific, Malaga, WA, USA) and air dried. Chromosomes were visualized by 10 minutes of staining in 5 % Giemsa solution (Sigma-Aldrich).

### Adipocyte differentiation

Py230 cells at approximately 70 % confluence were grown in complete medium containing 300 μM retinoic acid (Sigma-Aldrich) for seven to 10 days. The medium was then changed to complete medium with 5 μg/ml insulin (Sigma-Aldrich) for an additional seven to 10 days. 1 μM rosiglitazone (Sigma-Aldrich) added to the medium enhanced adipogenesis. Cells were fixed in 10 % neutral buffered formalin (Thermo Scientific), stained with oil red O (Sigma-Aldrich) and counterstained with methylene blue (Sigma-Aldrich).

### Immunofluorescence and immunohistochemistry

Cells plated onto tissue culture treated glass coverslips (Thermo Scientific) were fixed with methanol and stained with K8 (TROMA-1, Developmental Studies Hybridoma Bank, Iowa City, IO, USA) and K14 (Covance, San Diego, CA, USA) primary antibodies overnight at 4 °C. Coverslips were washed with PBS and secondary anti-rat Alexa488 or anti-rabbit Alexa647 (Life Technologies, Grand Island, NY, USA) added for one hour at room temperature (RT). After washing in PBS, the coverslips were mounted in Vectorshield with 4',6-diamidino-2-phenylindole (DAPI; Vector Laboratories, Burlingame, CA, USA) and viewed with a Zeiss 5Live line-scanning confocal microscope using a 1.2 N.A. water immersion 40X objective (Carl Zeiss MicroImaging GmbH, Jena, Germany). Images were captured using Zen software (Carl Zeiss MicroImaging). A total of 646 Py230 and 221 Py9813 cells from early passages, less than P5, were analyzed for the distribution of double-positive, single-positive and double-negative cells. Mammospheres were fixed in methanol and stained in suspension using the K8 and K14 antibodies described as above.

For immunohistochemistry (IHC), paraffin-embedded tissues were sectioned at 5 μm, deparaffinized in xylene and rehydrated through a series of alcohols. Antigen retrieval was carried out by autoclaving slides in citrate buffer pH 6.0 for 20 minutes. Slides were cooled to room temperature and endogenous peroxidases blocked with 0.3 % H_2_O_2_ in ice cold methanol for 30 minutes for estrogen receptor (ER) (MC-20, Santa Cruz, Dalla, TX, USA) and progesterone receptor (PR) (A0098, Dako, Carpinteria, CA, USA) staining or 3 % H_2_O_2_ in dH_2_O for 10 minutes for K5 (Abcam, Cambridge, MA, USA) or p53 (CM5, Vector Laboratories). To reduce non-specific binding, slides were incubated in 1 % BSA in PBS for 30 minutes. Antibodies in 1 % BSA in PBS were applied to the sections overnight. After washing off the antibody with PBS, anti-rabbit HRP (Vector Laboratories, 1:500) in 1 % BSA in PBS was added for 30 minutes. After thorough washing with PBS, slides were incubated with ImmPACT DAB reagent (Vector Laboratories) until the appropriate color reaction occurred. Slides were counterstained with hematoxylin (Vector Laboratories), dehydrated and mounted with cytoseal (Thermo Scientific).

### *In vivo* tumorigenesis and metastasis

To generate ER- and PR-positive tumors, 10^4^ or fewer Py230 cells were injected in 2 mg/ml matrigel into the thoracic or inguinal mammary fat pads of six to eight–week-old female mice. Tumors were removed and fixed in 10 % formalin for histologic analysis at 10 to 12 weeks following injection. For tumor heterogeneity experiments, 10^6^ Py230 cells were injected orthotopically. After six to eight weeks, the tumors were removed and enzymatically digested as described for primary tumors. Cells were washed with PBS and injected orthotopically into new hosts without any *in vitro* culture. The same procedure was repeated with T2 tumors to produce T3 tumors.

To examine the ability of small numbers of cells to form tumors, clonal Py230 cells were cultured for 48 hours in ultra-low adhesion plates, and then one or 10 cells picked by hand and placed in 10 μl 2 mg/ml matrigel, before being injected into the cleared mammary fat pad. Limiting dilution was used to prepare 100 or 1,000 cells for injection. For whole mounts of the mammary glands, normal adult, 16-day pre-lactating mammary gland, 12-week spontaneous hyperplastic mammary gland or cleared mammary fat pads injected with single-cell suspensions of Py230 cells at day 10 were removed and fixed in 10 % formalin. Whole mounts were stained with carmine using standard methods and imaged with an Olympus MVX10 macro-microscope (Olympus Imaging America, Center Valley, PA, USA) using diffuse, broadband white illumination.

Metastasis in the C57Bl/6 model requires sufficient time to allow tumor progression to occur. One to 10 cells gave rise to metastases over 20 to 24 weeks. For whole mounts, the mice underwent cardiac perfusion with 10 ml PBS, followed by intra-tracheal injection and lung expansion with 1 to 2 ml methyl Carnoy’s fixative. The lungs were stained with carmine solution using the standard whole mount procedure for mammary glands. To study the effects of estrogen on tumor growth, adult female mice were ovariectomized (OVX) at eight to 10 weeks of age. After two weeks of healing, tumor cells were implanted into the thoracic and inguinal mammary fat pads. Tumor growth was monitored with calipers as previously described, and tumors were weighed at the time of euthanasia. All animal studies including the use of tissue samples and cell lines were approved by the University of California, San Diego Institutional Animal Care and Use Committee under protocol S01162.

### Flow cytometry

Single-cell suspensions of tumors were prepared by enzymatic digestion as above. Briefly, tumors were dissociated with collagenase, red blood cells lysed, cell clumps digested with dispase (Stem Cell Technologies, Vancouver, Canada) and 100 μg/ml DNAseI (Sigma-Aldrich) and then trypsinized, and the resulting suspension was filtered through a 40-μm filter. The resuspended cells were stained with antibodies CD24, CD49f, CD29, CD44 and CD61 (Biolegend, San Diego, CA, USA), and analyzed on a BD FACAria (BD Biosciences, San Jose, CA, USA) using CellQuest software (BD Biosciences).

### Real-time polymerase chain reaction

Cultures were washed with PBS and RNA prepared using the RNeasy kit according to the manufacturer’s protocol (Qiagen, Valencia, CA, USA). First strand DNA was synthesized and RT-PCR carried out using Eva green mastermix in a StepOne Plus machine (Applied Biosystems, Foster City, CA, USA). Primers are listed in Table S1 (see Additional file 1). A thermal profile of 10 minutes at 95 °C, followed by 45 cycles of 30 seconds at 95 °C, 30 seconds at 60 °C and 30 seconds at 72 °C, with real-time fluorescence measured at the end of each annealing step was used. Melting curve analysis was performed on each run to confirm a single peak of activity for each primer pair.

### Assignment of breast cancer subtype to murine tumors by transcriptome profiling

RNAs were prepared using the RNeasy kit with on column DNAse digestion (Qiagen) according to the manufacturer’s protocol. RNA integrity was checked by electropherogram (Agilent Technologies, Santa Clara, CA, USA) and applied to Nimblegen expression arrays (Roche, Pleasanton, CA, USA). To match our MaCSC-derived syngeneic murine breast tumors to their most relevant human breast cancer subtypes based on the global similarities in gene expression pattern, we carried out transcriptome profiling on our murine tumors and then employed a supervised learning approach to assign the subtype labels to each murine tumor. First, we extracted 1,300 intrinsic genes that could be reliably measured across different microarray platforms for use in human breast tumor profiling [12]. We then used the ‘homologene’ database [13] to map these genes from human gene IDs to mouse gene IDs. Of the 1,300 human genes, 1,021 could be mapped to the mouse genes assayed by the Nimblegen microarray 12x135K, and our classifier was based on these shared genes between human and mouse. Next we assembled two independent human breast cancer datasets, with expression microarray data and intrinsic subtype annotations, one for training and the other for validation. The training dataset (N = 311) contained 73 basal-like, 100 luminal A, 62 luminal B, 39 Her2-enriched and 37 claudin-low subtypes [14]. The validation dataset (N = 511) contained 95 basal-like, 231 luminal A, 127 luminal B and 58 Her2-enriched subtypes [15]. Then a k-nearest neighbor classifier [16] was built on the training dataset, rigorously assessed by the validation dataset and finally applied to the mouse samples to classify them (Table S2, see Additional file 1). The data reported in this paper have been deposited in NCBI's Gene Expression Omnibus (GEO) database (accession number [GEO: GSE61138]) [17].

### Statistical analysis

Data are presented as mean ± standard error of mean (SEM). Differences between multiple groups were analyzed by analysis of variance followed by Sidak’s multiple comparisons test, using Prism software (GraphPad Software Inc., La Jolla, CA, USA). All results presented are representative of multiple experiments.

## Results

### Luminal tumors harbor multipotent mammary cancer stem cells

**Fig. 1:**
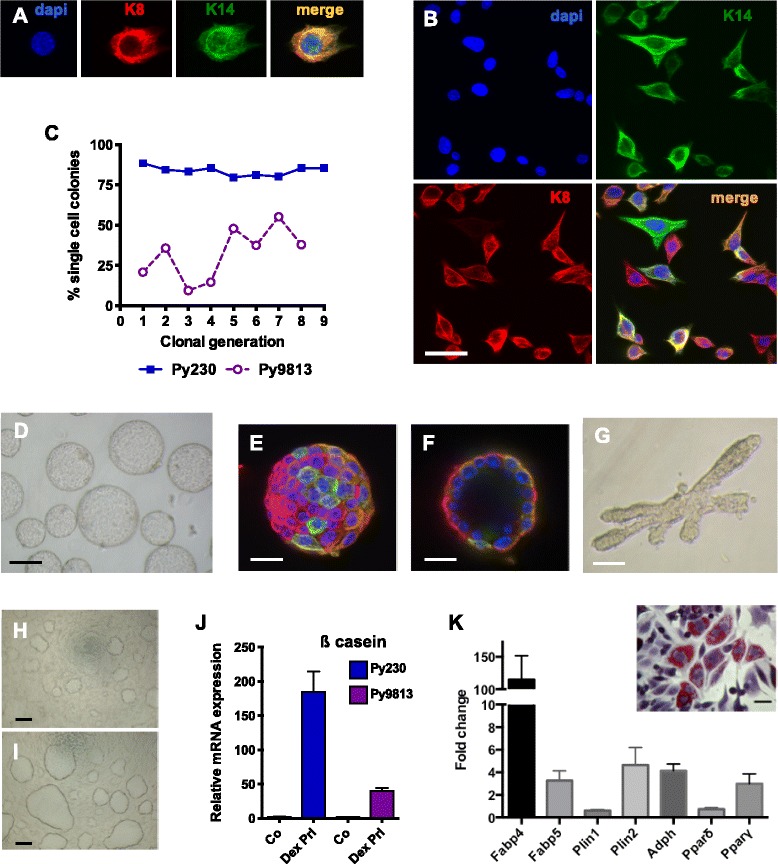
MaCSCs have the capacity to differentiate into multiple cell types. (**a**) A single Py230 cell stained with myoepithelial marker keratin 14 (K14) and luminal marker keratin 8 (K8). (**b**) Clonal Py230 cells grown on a glass coverslip stained with K14 and K8. Scale bar 20 μm. (**c**) Clonogenic efficiency of C57Bl/6 MaCSC line Py230 and FVB/N MaCSC line Py9813. (**d**) Hollow mammospheres of Py230 cells in suspension culture. Scale bar 50 μm. (**e,f**) Py230 mammosphere stained with K14 and K8. Scale bar 20 μm. (**g**) Py230 mammosphere grown on collagen exhibits branching structures. Scale bar 100 μm. (**h**) Confluent Py230 cells spontaneously form domes. (**i**) Domes become enlarged upon treatment with lactogenic hormones dexamethasone and prolactin. Scale bar 50 μm. (**j**) Expression of beta-casein by MaCSC lines Py230 and Py9813 following treatment with lactogenic hormones. Data are means ± SEM of triplicate samples. (**k**) Py230 cells treated with retinoic acid and rosiglitazone express genes associated with adipocyte differentiation. Data are means ± SEM of triplicate samples. Inset: Py230 cells stained with oil red O. Scale bar 10 μm

We first examined whether C57BL/6 Py230 and FVB/N Py9813 cells had the ability to differentiate into luminal, myoepithelial and alveolar lineages present in normal mammary glands. In two-dimensional culture, single cells of both cell lines co-express the luminal marker keratin 8 (K8) and the myoepithelial marker keratin 14 (K14), and give rise to single-positive K8 and K14 cells (Fig. 1a, b) as they expand in culture. Additional file 2: Figure S1A-F shows Py9813 and Py230 differentiation in more detail, with early clonal colonies consisting of only double-positive cells and single-positive cells arising as further proliferation takes place. The majority of cells are double-positive, indicating that double-positive single cells have the ability to self-renew as well as differentiate. There are significantly fewer single-positive than double-positive cells (*P* <0.0001), and most single-positive cells express K8 in keeping with the formation of luminal tumors in the transgenic PyVmT model (Additional file 2: Figure S1E). Thus cultures can be expanded and recloned for multiple generations, with the percentage of single cells giving rise to new colonies varying between cell lines (Fig. 1c).

Hollow mammospheres formed by the cells in suspension culture contain a mixture of double-positive and single-positive cells (Fig. 1d-f) and when placed on collagen gels, Py230 mammospheres can form three-dimensional branching structures that are reminiscent of embryonic mammary organ cultures (Fig. 1g [18]). Py9813 mammospheres do not branch *in vitro*. Confluent monolayer cultures of both cell lines spontaneously give rise to domes that represent an early stage of lactogenic differentiation and have previously been described as being secretory structures conserved between pre-lactating epithelial cells and mammary tumor cells [19, 20].

Upon stimulation with lactogenic hormones dexamethasone and prolactin, Py230-derived domes expand and beta casein expression is dramatically upregulated, consistent with differentiation into alveolar cells (Fig. 1h-j). Py9813 cells also upregulate beta casein, but to a lesser degree than Py230 cells. In addition to the potential to differentiate into the mammary lineages, treatment of Py230 or Py9813 cells with adipogenic agents results in adipocyte differentiation and an increase in adipocyte-associated gene expression (Fig. 1k). Origins and MaCSC properties of the cell lines are summarized in Table S3 (see Additional file 1).

### Multipotent mammary cancer stem cells form luminal tumors and metastasis

**Fig. 2:**
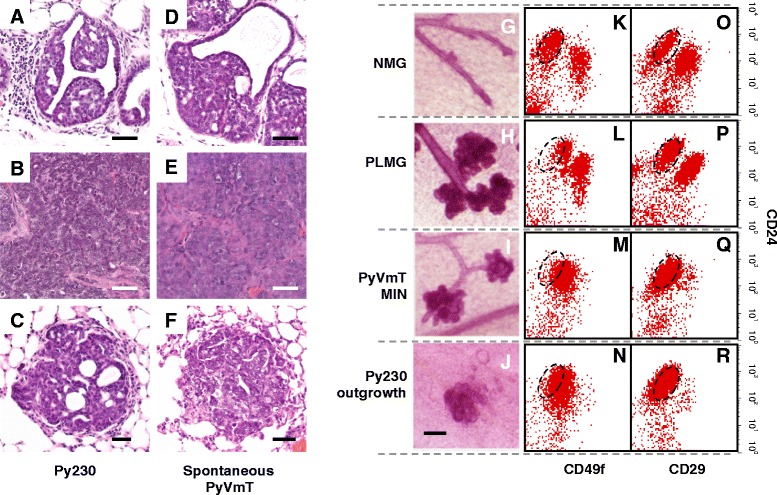
Py230 mammary cancer stem cells recapitulate spontaneous luminal tumorigenesis and metastasis. **a-f**, H&E stained sections of Py230 MIN (**a**), tumor (**b**) and metastasis (**c**) compared with spontaneous PyVmT MIN, (**d**), tumor (**e**) and metastasis (**f**). Scale bar = 50 μm. **g-j** Whole mounts of normal mammary gland (NMG), pre-lactating mammary gland (PLMG), PyVmT MIN and a 10-day outgrowth of Py230 tumor cells in the cleared mammary fat pad. Scale bar = 100 μm. **k-r**, Representative dot plots of NMG (**k,o**), PLMG (**l,p**), PyVmT MIN (**m,q**) and Py230 tumor (**n,r**) labeled with CD24 and CD49f (**k-n**) or CD24 and CD29 (**o-r**)

When implanted into the mammary fat pads of syngeneic immune competent C57Bl/6 mice, Py230 cells form mammary intraepithelial neoplasia (MIN), from which a subset of cells develop into well-differentiated carcinomas and then poorly differentiated carcinomas that metastasize to the lungs (Fig. 2a-c). While a transplantation model of premalignant PyVmT FVB/N cells forms MIN [21], Py9813 cells do not form MIN, but do form luminal tumors when implanted orthotopically. By standard hematoxylin and eosin (H&E) histology, the MIN, carcinomas and metastases that form upon Py230 injection are morphologically identical to those present in the spontaneous C57Bl/6 model (Fig. 2d-f). Whole mount analysis indicates that in comparison to normal, pre-lactating or hyperplastic mammary glands, Py230 outgrowths in the cleared mammary fat pad resemble the TDLU (Fig. 2g-j). Since no elongated branching ductal structures are formed in the orthotopic site to enable the outgrowths to fill the mammary fat pad, it appears that the Py230 cells are capable of forming branching ductal structures under certain conditions *in vitro*, with these structures being limited in length in the more complex *in vivo* microenvironment.

Injection of limiting cell numbers into the cleared mammary fat pad confirmed that a single Py230 cell can recapitulate luminal mammary tumorigenesis and lung metastasis (Figure S2A-D, see Additional file 3). As indicated by immunostaining for the myoepithelial protein keratin 5 (Figure S2A, see Additional file 3), the proportion of myoepithelial cells in Py230 outgrowths is low, indicating a bias towards development of luminal tumor cells, consistent with multiple studies indicating that PyVmT oncogenic pathways form luminal tumors. The differentiation and biologically appropriate organization of Py230 cells into both myoepithelial and luminal lineages in the absence of endogenous mammary epithelium, confirms their tissue stem cell-like differentiation potential and identifies them as mammary CSCs (MaCSCs). Furthermore, 100 % of the 36 subclones injected in the course of the Py230 clonal ability analysis formed luminal tumors, as observed by H&E staining. Py9813 was slightly less robust, with 70 % of clones tested forming tumors.

The luminal profile of both spontaneous PyVmT tumors and Py230-derived tumors was confirmed by flow cytometry using standard markers that define luminal and basal subpopulations in the normal mammary gland (Fig. 2k, o) [22, 23]. The expansion of the TDLU observed in PyVmT MIN (Fig. 2i) is very similar to the expansion of the TDLU in the pre-lactating mammary gland (Fig. 2h), and there is a concomitant increase in the level of α6 integrin as measured by CD49f expression, in the cell population containing luminal progenitors (gated) compared with normal mammary gland, that may reflect the proliferative state of the cells (Fig. 2k-m) [24].

### Relationship to heterogeneous human breast cancer

**Fig. 3:**
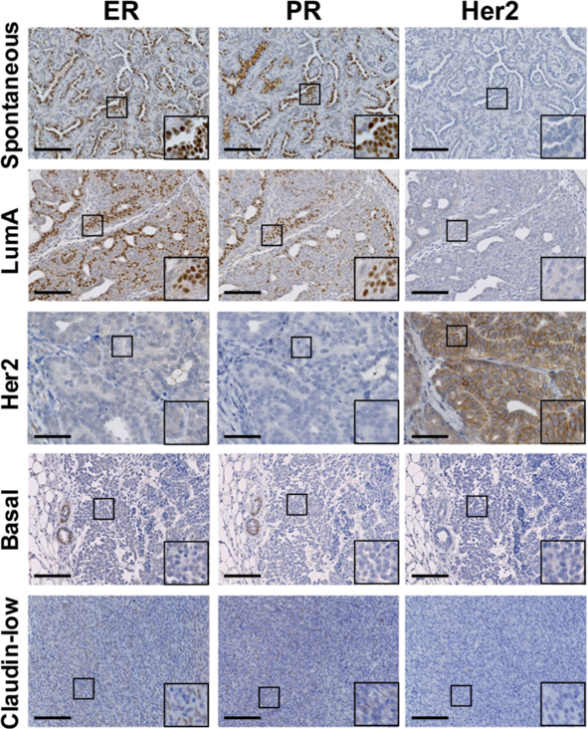
Py230 cells differentiate to form multiple tumor subtypes *in vivo*. Spontaneous PyVmT tumors (upper panels) or Py230-derived tumors representing different subtypes: luminal A, Her2, basal and claudin-low (lower 4 panels) stained for estrogen receptor (ER), progesterone receptor (PR) or Her2 expression

Human tumors have been classified into five major subtypes: luminal A, luminal B, Her2 overexpressing, basal-like and claudin-low. Genomic analysis of mouse models of breast cancer indicates that the combination of individual models can recapitulate human breast cancer heterogeneity [25], but to date no single model has demonstrated this ability. We have found that in contrast to FVB/N PyVmT tumors that are hormone receptor-negative and classify with human luminal B tumors by genetic analysis [26], spontaneous PyVmT tumors in the C57BL/6 background, which have a significantly longer tumor latency [9], can form hormone receptor-positive tumors (Fig. 3). In concordance with the transgenic models, Py9813 FVB/N cells do not give rise to hormone receptor-positive tumors, but Py230 cells that are ER- and PR-negative under normal *in vitro* culture conditions, can upregulate ERα expression in estrogen-depleted media, and can form ER- and PR-positive tumors *in vivo* (Fig. 3, Figure S3A, B, see Additional file 4). Interestingly, Py230 cells only give rise to hormone receptor-positive tumors when injected orthotopically at relatively low cell densities (1 x 10^4^ cells or less), suggesting that the formation of hormone receptor-positive tumors may result from paracrine signaling between normal mammary cells and tumor cells. Orthotopic injections into 10 cleared mammary fat pads at low cell densities did not form hormone receptor-positive tumors. In concordance with a female hormone responsive phenotype, Py230 tumor growth is compromised in ovariectomized mice (Figure S3C-F, see Additional file 4).

**Fig. 4:**
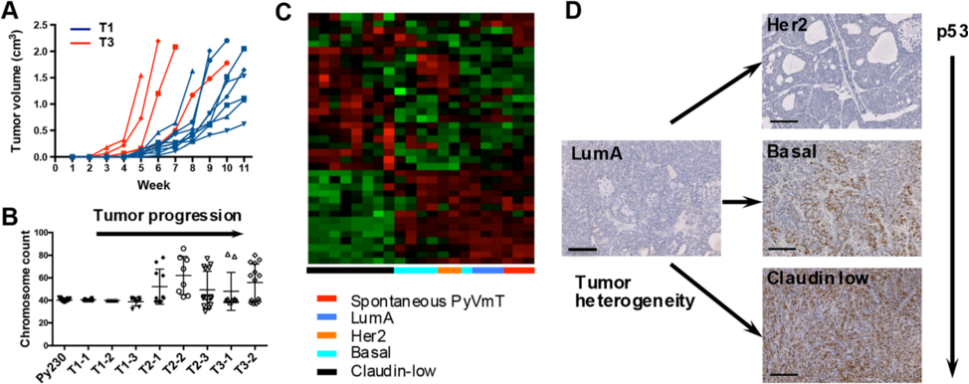
During tumor progression Py230 cells undergo changes that upregulate nuclear p53 expression. (**a**) Tumor latency of Py230 tumors from first generation tumor cell injections (T1, blue) compared with Py230 tumors from third generation injections (T3, red). (**b**) Karyotyping of the Py230 cell line (Py230) and Py230 first generation tumors from independent animals (T1). Cells isolated from tumors of subsequent generations (T2, T3) undergo changes that result in aneuploidy. (**c**) Expression profiling of tumors from various generations of Py230 injections compared with spontaneous PyVmT tumors. (**d**) IHC staining for p53 expression in the different Py230 tumor subtypes

To further understand the cellular basis for breast cancer heterogeneity, we explored whether clonal Py230 MaCSCs could give rise to breast cancer subtypes other than the luminal subtype from which they were isolated. The currently accepted definition, based in part on the properties of known cancer stem cells, posits that a cancer stem cell need only differentiate into the heterogeneous lineages of cancer cells that comprise the tumor of origin [27]. We found that in addition to forming luminal tumors, Py230 cells could be passaged *in vivo* in the immune competent mammary fat pad without intervening culture to form additional tumor subtypes including Her2, triple-negative basal-like and claudin-low tumors (Fig. 3), suggesting that different tumor subtypes may result from changes occurring during tumor progression. We also noted that tumors grew more rapidly in subsequent generations (Fig. 4a) and underwent genetic changes resulting in aneuploidy (Fig. 4b). These changes are not typically observed in the transgenic primary tumor model, as the mice develop large multifocal luminal tumors that necessitate euthanizing the mice before these genetic and epigenetic alterations take place.

To compare the global similarities of our MaCSC-derived syngeneic murine breast tumors with human breast cancer samples, our strategy was: to obtain a high quality microarray training dataset of human breast tumors of known intrinsic subtypes; to map the set of robust genes onto mouse gene homologues; to reclassify the human breast cancer training data into its intrinsic subtypes, using the human/mouse homologue genes, in order to develop the classification rule; and finally to use the trained classifier to classify the mouse tumors into these intrinsic subtypes. Therefore a KNN classifier (K = 13) was trained using 311 training samples. We assessed the performance of our KNN classifier using leave-one-out cross-validation (LOOCV), as well as an independent validation dataset. Our classifier could classify the 311 training samples into their subtypes with an overall accuracy of 87 % in LOOCV. When applied to an independent validation dataset, the overall accuracy is 84 %, comparable to that of LOOCV (Table S2, see Additional file 1). Therefore, we concluded that this gene expression based classifier has been well trained for predicting breast cancer subtypes. We then used this classifier to predict the subtype of 21 murine breast tumors we harvested and these were classified as six luminal A, two Her2-enriched, five basal-like and eight claudin-low.

To provide further evidence that our classification of the murine tumors truly reflects their similarities to the human counterpart of breast cancer, we conducted Gene Set Enrichment Analysis (GSEA) [28] to compare between luminal A, basal-like and claudin-low murine tumors using established human gene signatures that are known to differ between human breast cancer subtypes. First, human claudin-low tumors are known to lack cell-cell junction proteins. We downloaded the gene set ‘REACTOME_CELL_CELL_JUNCTION_ORGANIZATION’ from the MsigDB database [28] and found this gene set, as expected, is significantly downregulated in the claudin-low murine tumors (*P* <0.001) as compared to either luminal A or basal-like murine tumors.

Second, we confirmed that the expression patterns of our murine subtypes are highly consistent with those of human breast cell lines [29]. The gene signatures enriched in luminal, basal and mesenchymal human cell lines [29] are each enriched in our luminal A, basal-like and claudin-low murine tumors, respectively. The *P* values for the cell line signatures are: luminal A versus claudin-low, 0.002; basal-like versus claudin-low, 0.006; and luminal A versus basal-like, 0.02. Finally, Lim *et al*. [30] have developed conserved (between human and mouse) expression signatures for two normal cell subpopulations (mammary stem cell enriched versus luminal progenitor) isolated from the mouse mammary gland. We hypothesize that our claudin-low tumors resemble their mammary stem cell-enriched population, while our luminal tumors resemble their luminal progenitor population. GSEA analysis using their expression signatures confirmed this hypothesis (Figure S4A-F, see Additional file 5).

For the purpose of visualization and biological interpretations, we also developed a gene signature with only dozens of genes solely from the human training samples. Briefly, for each subtype we used the limma approach [31] to select the 10 most differentially expressed genes (comparing each subtype versus the combined other subtypes). We took the union of the selected genes over the subtypes to form a 37-gene expression signature (as some genes were not unique to a single subtype; Table S4, see Additional file 1). We did an unsupervised hierarchical clustering of our murine tumors using the 37-gene expression signature. Figure 4c shows that mouse samples were nicely clustered according to their predicted subtypes, which were determined by the classifier using approximately 1,000 genes, demonstrating the expression similarity within each subtype and obvious differences between subtypes. Validation of gene expression by semi-quantitative RT-PCR of a subset of key genes from the 37-gene signature panel revealed considerable variability in expression between different epithelial tumors, but a distinct difference between the epithelial and claudin-low tumors, which were more mesenchymal in phenotype (Figure S4G, see Additional file 5).

### Tumor progression is associated with p53 dysregulation

**Fig. 5:**
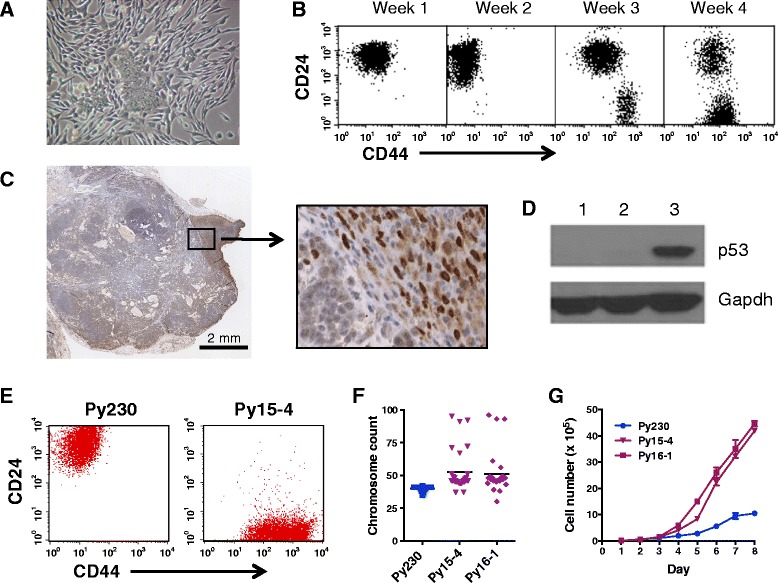
Py230 cells differentiate to form p53-positive mesenchymal cells. (**a**) Py230 MaCSCs treated with 0.1 % DMSO undergo epithelial to mesenchymal transition. (**b**) Flow cytometry profiles of a DMSO-treated Py230 clone showing the transition over time from a luminal to a claudin-low phenotype. (**c**) A third generation Py230 tumor showing clonal evolution to form an invasive p53-positive tumor extension. (**d**) Western blot of tumor lysates. Lane 1: tumor from early passage Py230 cells, lane 2: basal-like tumor from Py117, a cell line cloned from a third generation *in vivo* passaged Py230 tumor, lane 3: claudin-low tumor from Py15-4, a cell line clone from a third generation *in vivo* passaged Py230 tumor. (**e**) CD24, CD44 FACS profile of Py230 and Py15-4 clonal cell lines. (**f**) Karyotype of clonal luminal cell line Py230 and clonal claudin-low cell lines Py15-4 and Py16-1. (**g**) Growth curves of Py230 luminal cell line and Py15-4 and Py16-1 claudin-low cell lines. Data are means ± SEM of triplicate cultures. Both claudin-low cell lines were derived from Py230 cells

Breast cancer progression in humans is associated with the development of aggressive triple-negative and/or basal-like breast tumors that have dysregulated p53 activity resulting in nuclear accumulation of p53. In concordance we found in IHC studies that Py230-derived tumors underwent genetic and/or epigenetic changes that resulted in increased nuclear p53 expression indicative of dysregulation of the p53 pathway (Fig. 4d). Since the IHC data suggested dysregulation of the p53 pathway following serial tumor passaging, we investigated if a gene set consisting of direct p53 effectors (‘PID_P53DOWNSTREAMPATHWAY’ from the MsigDB database) is differentially expressed between luminal A and claudin-low murine tumors. In agreement with our IHC results, this pathway is significantly downregulated in claudin-low tumors (*P* <0.05, Figure S5A, B, see Additional file 6), probably as a consequence of p53 malfunction. RT-PCR analysis of p53 pathway genes confirmed a significant downregulation of the pathway in Py230-derived claudin-low tumors compared with early generation Py230 luminal tumors. To determine whether Py230 cells could give rise to cells with a mesenchymal phenotype *in vitro*, luminal Py230 cells were exposed to the differentiation agent DMSO (Fig. 5a). Within 48 hours, cells with a mesenchymal phenotype could be seen migrating from the cobble-stone cluster of Py230 cells. Flow cytometry studies showed the change from a luminal CD24hiCD44lo population towards a CD24loCD44hi claudin-low population over time (Fig. 5b). IHC staining of tumors and Western blotting proteins from cell lines derived from *in vivo* passaging of Py230 confirmed that mesenchymal cells expressed nuclear p53 (Fig. 5c, d).

To examine the MaCSC properties of the Py230-derived mesenchymal cells, we performed flow cytometry on cell lines cloned from early and late generation Py230-derived tumors. The results suggest that CD24loCD44hi Py15-4 cells with a mesenchymal phenotype characteristic of cells currently described as breast cancer stem cells can be derived from CD24hiCD44lo luminal epithelial Py230 cells (Fig. 5e, Figure S5C, see Additional file 6). It is not surprising that our Py230 luminal MaCSCs do not express CD44, a marker commonly used to identify breast cancer stem cells that give rise to aggressive triple-negative breast cancers [2, 32, 33].

The clonal claudin-low cell lines that have dysregulated p53 activity have an aneuploid karyotype, grow significantly faster than cell lines that form luminal tumors (Fig. 5f, g) and have reduced differentiation potential (Figure S5D, E, see Additional file 6). CD24loCD44hi cells have no dome-forming ability or ß-casein expression in response to stimulation with lactogenic hormones *in vitro*. Gene array analysis of these cell lines indicated that genes associated with mammary luminal progenitor cells are enriched in the luminal Py230 MaCSC line compared with the claudin-low MaCSC line Py16-1 (Figure S4A, D, see Additional file 5), in concordance with data that Brca1-deficient mammary tumors also arise from luminal progenitor cells [34].

Since CD24loCD44hi cells are described as MaCSCs, we confirmed that expression of genes associated with the normal mammary stem cell phenotype are upregulated in claudin-low MaCSC line Py16-1 relative to luminal MaCSC line Py230 (Figure S4B, E, see Additional file 5) [30]. Furthermore, genes downregulated in mammary stem cells are upregulated in Py230 cells compared with Py16-1 cells (Figure S4C, F, see Additional file 5), confirming that luminal tumors arise from cells with a luminal progenitor profile, not a mammary stem cell profile.

## Discussion

**Fig. 6:**
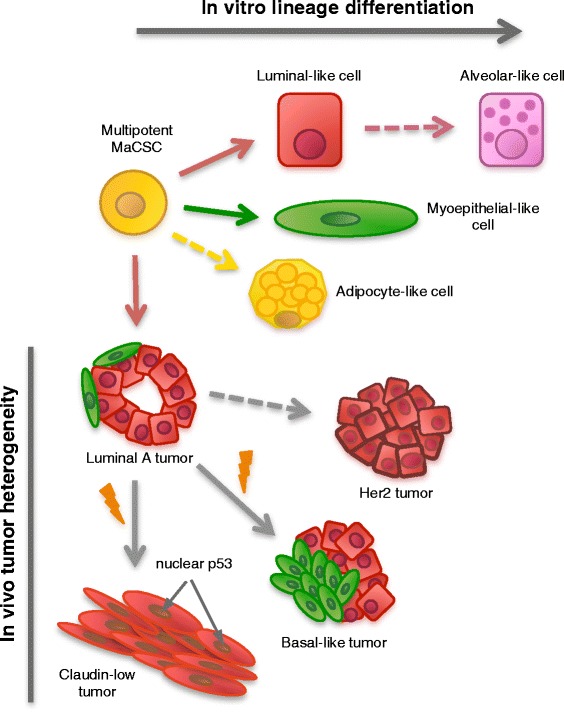
Py230 MaCSC model. Py230 MaCSCs undergo lineage differentiation *in vitro* in response to appropriate stimuli. We postulate that the alveolar-like cells develop from luminal cells and that adipocyte-like cells differentiate from the multipotent MaCSC, hence the dashed lines. *In vivo*, Py230 MaCSCs consistently form luminal tumors. With serial passaging *in vivo*, epigenetic and/or genetic changes taking place during tumor progression that can lead to clonal evolution and tumor heterogeneity. We have derived clonal cell lines that give only to claudin-low tumors or basal-like tumors. To date we have not isolated clonal cell lines that give rise to Her2 tumors

Plasticity is an important, but complex property of breast cancer stem cells that impacts the type of tumor formed. Currently, only a small proportion of aggressive human breast cancers are thought to arise from breast CSCs [2, 35]. In this study, we show that luminal tumors, which form the majority of human tumors, can indeed be formed by multipotent MaCSCs. The MaCSCs that form luminal tumors can differentiate into all mammary lineages and form a range of tumor subtypes relevant to human breast cancer (Fig. 6), suggesting a need to reevaluate our current understanding of the cells that may be involved in human breast cancer development and progression. Orthotopic injection of a single MaCSC can result in tumor formation and metastasis in an immune-competent host, establishing the clinical relevance of this model for preclinical therapy studies. Our studies confirm and extend previous work showing that PyVmT lentiviral transduction of mixed lineage mammary epithelial cells can generate tumors with diverse histological and molecular subtypes [36], that precancerous mammary intraepithelial neoplasia outgrowth MINO tissues are programmed for carcinogenesis and metastasis [37], and that cell lines producing either epithelial or myoepithelial-like cells can be isolated from PyVmT tumors [38].

Previous reports have shown that the majority of normal murine mammary stem cells capable of repopulating the cleared mammary fat pad reside in the basal CD24loCD49fhiCD29hi cell subpopulation of normal mammary epithelium [22, 23]. By contrast, PyVmT MIN and tumors show preferential expansion of the luminal progenitor CD24hiCD49intCD29lo subpopulation, suggesting that mammary tumors arise from a different cellular compartment than normal mammary epithelium. Whether this is the result of the reprogramming of mature luminal or luminal progenitor cells to a more stem-cell-like state [39] is unknown, however, the clonal cells giving rise to luminal tumors do co-express the bipotential mammary stem cell markers K8 and K14.

It has long been recognized that the stromal composition of human and murine mammary gland differs significantly, with a higher fibroblastic component in normal human mammary glands and breast cancers. Thus the previous inability to generate hormone receptor-positive tumors in mice may have been due to a lack of understanding of the cells and conditions required for this process, rather than an innate species difference. The slower development of mammary tumors in the C57Bl/6 background compared with the FVB/N background [9] may allow additional tumor-stromal cell and/or tumor-epithelial cell interactions to take place.

Studies in developmental biology and tumor biology have shown that a number of the genes associated with stem cells, such *cadherin1* and *snail2*, are also genes associated with epithelial to mesenchymal transition (EMT) in differentiated cells. Our data support the premise that compared with the luminal MaCSCs, the claudin-low MaCSCs are more differentiated cells that have undergone EMT. Since both cell lines comply with the current definition of a CSC [27], but Py230 and similar MaCSC lines have more mammary cell lineage differentiation potential under our assay conditions than claudin-low MaCSC lines such as Py16-1, we raise the possibility that a hierarchy of MaCSCs exists, and that Py230 cells are the primary MaCSCs from which multiple subtypes of tumors and CSCs can be derived. An alternative hypothesis is that the Py230 cells dedifferentiate into a more stem-cell like, claudin-low cell type. While the expression data would support this hypothesis, the karyotyping indicating that the claudin-low cell population derived from Py230 cells is aneuploid, suggests this is not the case in our model system. Unlike normal stem cell differentiation in which cells maintain a normal karyotype and cell fate is predetermined, we cannot currently predict the exact tumor subtypes arising from the differentiation of Py230 cells *in vivo*. Whether the subtypes are imposed purely by stochastic genetic aberrations or in part, by specific driver mutations occurring during tumor progression requires further investigation. However, it is clear that while transcriptional program changes promote normal stem cell differentiation, genetic changes appear to be critical for Py230-derived tumor heterogeneity (Fig. 6). Thus, in our model system, luminal tumors arise from a multipotent MaCSC, while tumor heterogeneity appears to be derived from clonal evolution. In concordance, we have additional clonal cell lines with differentiation properties intermediate to those of the luminal and claudin-low MaCSCs. These MaCSC lines, such as Py117, give rise to triple-negative basal-like tumor subtypes. Moreover, we have not observed a progression from Her2 to basal-like then to claudin-low, rather each tumor subtype appears to arise directly from the luminal A-type tumor.

A recently described taxonomy of breast cancer emphasizes similarities between normal breast cell types and human breast tumors [40], suggesting that our murine model system has important parallels with human breast cancer. In addition to the MaCSCs isolated from PyVmT mice in a wild-type background, we have also isolated MaCSCs from PyVmT mice harboring gene-targeted mutations, suggesting that our cell lines will be useful in further identifying factors important in breast cancer initiation and progression.

## Conclusions

Human breast cancer is characterized by tumor heterogeneity. We have identified a new class of clonal multipotent MaCSCs that can give rise to luminal tumors; the most common subtype of human tumor. A single MaCSC can give rise to metastasis in the immunocompetent host. Tumor evolution *in vivo* gives rise to more aggressive tumor subtypes that can have dysregulated p53, and characterization of MaCSCs from the different tumor subtypes suggests a developmental hierarchy of MaCSCs. These MaCSCs may help to link the stem cell and clonal expansion theories of breast cancer progression.

## Additional files

Additional file 1: Table S1. Oligonucleotides. **Table S2.** Confusion matrix for the validation dataset. **Table S3.** Cell line properties. **Table S4.** 37 Geneset. (PDF 102 kb) Additional file 2: Figure S1. Clonal MaCSC differentiation. **A**. Clonal single cells from FVB/N cell line Py9813 are K8K14-positive. Left panel stained with secondary antibody alone. **B**. Early colonies contain only double-positive cells. Scale bar 20 μm. **C**. In two-dimensional cultures, K8 + K14+ Py9813 cells can self-renew or differentiate to form single-positive K8 or K14-positive cells. Scale bar 20 μm. **D**. Py9813 cells form hollow mammospheres that show the presence of double-positive cells and K8 single-positive luminal cells. Single-positive myoepithelial cells are less commonly observed. Scale bar 20 μm. **E**. The percentage of double-positive, single-positive and double-negative cells in immunofluorescence-stained two-dimensional cultures. Analysis of variance showed the groups were highly significantly different, *P* <0.0001 and by Holm-Sidak’s multiple comparisons test, there were significantly fewer K8+ and K14+ cells compared with K8 + K14+ cells, *P* <0.0001, and significantly fewer K14+ cells than K8+ cells, *P* <0.01 in both cell lines. No double-negative cells were observed in cultures of either cell type. Data are means ± SEM. **F**. Cultures of control or 300 nM retinoic acid-treated Py9813 cells stained with oil red O. Scale bar 20 μm. (PDF 900 kb) Additional file 3: Figure S2. Py230 MaCSCs recapitulate spontaneous tumorigenesis and metastasis. **A**. Left panel - A Py230 MIN outgrowth in the cleared mammary fat pad. Scale bar = 2 mm. Right panel - Higher magnification showing K5-positive cells (brown) lining the hyperplastic ductal structures. Scale bar = 100 μm. **B**. Tumor formed from a single Py230 cell injected into the cleared mammary fat pad of a three-week-old mouse. At 29 weeks the tumor showed microcystic and solid areas of adenocarcinoma. Scale bar 200 μm Lung metastases indicated by the white arrows were observed in whole mounts of the lung. Inset shows H&E-stained lung metastasis. Scale bar 100 μm. **C**. Lung metastases (black arrows) visualized in whole mounts of a lung from a wild-type mouse with a 10-cell Py230 tumor. Inset shows H&E-stained lung metastasis. Scale bar 100 μm. **D**. Percentage of tumors formed by varying numbers of Py230 cells injected in 2 mg/ml matrigel into the cleared mammary fat pads of three to four-week-old female mice. (PDF 911 kb) Additional file 4: Figure S3. Py230 cells are estrogen responsive. **A**. Py230 cells cultured on glass coverslips for 48 hours in F12K/DMEM media with 5 % fetal calf serum. **B**. Py230 cells cultured on glass coverslips for 48 hours in F12K/DMEM media without phenol red with 5 % charcoal-treated serum. **C, D**. 10^4^ × Py230 cells injected into intact or OVX female mice were monitored over an eight-week period. Tumor volume and final tumor burden data indicated that the majority of Py230 tumors were sensitive to the presence of endogenous estrogen. Data are means ± SEM from 16 tumors in four mice per group. **E,F**. Tumors from the same dataset that have undergone epigenetic and/or genetic changes resulting in increased growth (encircled in **D**) have been removed. Data are means ± SEM from 15 tumors in intact mice and 13 tumors in OVX mice. Data are representative of two similar experiments. (PDF 504 kb) Additional file 5: Figure S4. Validation of expression array data by GSEA and RT-PCR. **A**. Gene set for mammary luminal progenitor cells is significantly enriched (*P* <0.001) in luminal versus claudin-low tumors. **B**. Gene set for mammary stem cells is not enriched in luminal versus claudin-low tumors. **C**. Genes downregulated in mammary stem cells are significantly enriched (*P* <0.01) in the Py230 luminal cell line compared with the Py16-1 claudin-low cell line. **D**. Confirmation of lower expression of several mammary luminal progenitor-associated genes in Py16-1, a claudin-low cell line compared with the parental Py230 cell line. **E**. Confirmation of higher expression of several mammary stem cell-associated genes in Py16-1, a claudin-low cell line compared with the parental Py230 cell line. **F**. Higher expression of genes downregulated in mammary stem cells in luminal Py230 cell line compared with claudin-low Py16-1. For each gene comparison **P* <0.05, ****P* <0.005, *****P* <0.001 with Sidak’s correction for multiple comparisons, following two-way analysis of variance. Data are means ± SEM for three independent cultures per group. **G**. 35 independent tumors were examined for expression of each of the indicated markers. The results were compared with expression by normal prelactating mammary gland (PLMG), which is the normal control for highly proliferative mammary tumors and spontaneous PyVmT tumors (*n* = 4 per group). (PDF 386 kb) Additional file 6: Figure S5. Divergent mammary cancer stem cell phenotypes. **A**. Enrichment plot of p53 pathway genes in LumA or Claudin-low MaCSCs. B. Real-time PCR validation of the seven most highly differentially expressed genes in the p53 pathway analysis. Data are means ± SEM of six independent tumor samples per group. All groups were significantly different at *P* <0.005 or more using the Holm-Sidak correction for multiple comparisons. **C**. EMT-associated gene expression in Py230 and Py15-4 cell lines. **D**. Confluent cultures of Py230 and Py15-4 MaCSCs treated for 48 hours with dexamethasone and prolactin. Arrowheads indicate dome formation. **E**. Relative expression of ß casein in Py230 and Py15-4 cultures treated for 48 hours with dexamethasone and prolactin. (PDF 476 kb)

## Abbreviations

DMSO: dimethyl sulfoxide
ER: estrogen receptor
MaCSC: mammary cancer stem cell
MINO: mammary intraepithelial neoplastic outgrowth
LOOCV: leave one out cross-validation
PR: progesterone receptor
PyVmT: polyomavirus middle T
TDLU: terminal ductal lobular unit
