# Supplementary material for: Multipotent luminal mammary cancer stem cells model tumor heterogeneity
Source: Breast Cancer Res. 2015 Oct 14;17:137. doi: 10.1186/s13058-015-0615-y (PMC4606989; doi:10.1186/s13058-015-0615-y)
Supplement: Additional file 1: Table S1. — Oligonucleotides. Table S2. Confusion matrix for the validation dataset. Table S3. Cell line properties. Table S4. 37 Geneset. (PDF 102 kb) [file 13058_2015_615_MOESM1_ESM.pdf]

Supplemental Experimental Procedures

Table S1. Oligonucleotides

Gene	GenBank-EMBL number	Primer 1 (forward)	Primer 2 (reverse)
GAPDH	NM_008084	AGGTCGGTGTGAACGGATTTG	TGTAGACCATGTAGTTGAGGTCA
ß-actin	NM_007393	GGCTGTATTCCCCTCCATCG	CCAGTTGGTAACAATGCCATGT
ß casein	NM_009972	GGCACAGGTTGTTCAGGCTT	AAGGAAGGGTGCTACTTGCTG
Bcl214	NM_025778	TGGCCTTCTACGCCAGACA	CCAGGAATCAGTTGACCAAGTC
Bgt2	NM_007570	ATGAGCCACGGGAAGAGAAC	GCCCTACTGAAAACCTTGAGTC
Ccnd2	NM_009829	GAGTGGGAACTGGTAGTGTTG	CGCACAGAGCGATGAAGGT
Cdh1	NM_009864	GCCATTGCCAAGTACATCCT	TATCTCCATGGGATCCTCCA
Cldn7	NM_001193619	GGAGACACTGACAGGCTCAGGTGC	GGCGACCCGCTTCACCGCTC
Csn3	NM_007786	ATTCTGGCATTAACTCTGCCC	AAAGATGGCCTGTAGTGGTAGTA
Epcam	NM_008532	TCAGAGCTTGCAGACTGCGCT	TCGAGCGGCTCTCCGTTCACT
Elf5	NM_010125	CTGGGAGCGCGCTTGCCTTC	TCGGTCCAAGGCATCAGGGGG
ER alpha	NM_007956	TGCACCATTGACAAGAACCGGA	AGCACCCATTTCATTTCGGCCT
ErbB2	NM_001003817	GGCACTGTCTACAAGGGCAT	GAGGCGGGACACATATGGAG
Esrp1	NM_194055	TGGTGCTTTTTGGGATCACTG	CCACGACTTTCCATAACAGCAG
Fabp4	NM_024406	GGATTTGGTCACCATCCGGT	TTCACCTTCCTGTCGTCTGC
Fabp5	NM_010634	ACGGGAAGGAGAGCACGATA	GAGTGCAGGTGGCATTGTTC
Foxa1	NM_008259.3	TGGCTCCAGGATGTTAGGGA	GTGTCCGCGTAGTAGCTGTT
Foxi1	NM_023907	CGAAGGTGGTGGCGAATAC	TGAGGGCTGAATAGGAATATGGT
Gata3	NM_008091	AGGCAACCACGTCCCGTCCT	TACCTGGCTCCCGTGGTGGG
Grhl1	NM_001161406	AGGGTGCAAGTGCTGAAAAAC	TGGCCTCTCTTATCAATGCCC
Hif-1α	NM_010431.2	GAAATGGCCCAGTGAGAAAA	CTTCCACGTTGCTGACTTGA
Hivep3	NM_010657	AGAGGCCATTCAGACGAGTGT	CTGCGGAAGCTGAGAGATGT
Kit	NM_001122733	GCCTGACGTGCATTGATCC	AGTGGCCTCGGCTTTTTCC
Lgals1	NM_008495	GTGCCTACACTTCAATCCTCG	GTTCCCGGTGTTCGGTTCC
Mmp2	NM_008610	TGGCAGCCCATGAGTTCGGC	TCGGGGGAGGGCCCATAGAG
Noxo1	NM_027988	GCTCCATTGCTGACACGTC	AGGTTTGGGTACAAAGAAGCC
Nrg1	NM_178591	ATGGACAGCAACCCAAGTTC	CTGCTGGGTTAGTCCTGCTC
Perp	NM_022032	ATCGCCTTCGACATCATCGC	CCCCATGCGTACTCCATGAG
Perilipin1	NM_175640	GAGTGTGGGGTCCTTGGGCGT	TCACAGGTCCCGCTCACCACA
Perilipin2	NM_007408	TGCGGCCATGACAAGTGCCC	CGCCCCAGTTACGGCACCTC
PPAR-δ	NM_011145	ATGGGACTCACTCAGAGGCT	TGGCTGTTCCATGACTGACC
PPAR-γ	NM_001127330	CCCAGAGCATGGTGCCTTC	AGTTGGTGGGCCAGAATGGCA
Ppp1r13b	NM_011625	TCCCATCCCCTATGACCACAT	TTGTTCACTGCTCTCAGTTGG
PR	NM_008829	AGGTCTACCCGCCATACCTT	CTTGCAGCTCCCACAGGTAA
Rb1	NM_009029	TGCATCTTTATCGCAGCAGTT	GTTCACACGTCCGTTCTAATTTG
SerpinB5	NM_009257	GCCCTGAGACTGGCAAATTCA	GCGCAAGGGACAGAGAAGTA
Snai1	NM_011427.2	CTACCGACCTTGCGCGACCC	AGGTGAACTCCACACACGCGT
Snai2	NM_011415	CTGGACCGTTATCCGCCCGC	TGGAGTGGAGCTGCCGACGA
Twist	NM_011658.2	CCACGAGCGGCTCAGCTACG	AATGACATCTAGGTCTCCGGCCTGC
Vdr	NM_009504	ACCCTGGTGACTTTGACCG	GGCAATCTCCATTGAAGGGG
Vim	NM_011701	CGTCCACACGCACCTACAG	GGGGGATGAGGAATAGAGGCT

Supplemental Information

Table S2. Confusion matrix for the validation dataset (N=511)

	Basal like **	HER2 **	Luminal A **	Luminal B **
Basal like *	**94**	1	0	0
HER2 *	0	**39**	1	18
Luminal A *	0	2	**210**	19
Luminal B *	0	2	37	**88**

* annotated human breast cancer subtypes. ** predicted human breast cancer subtypes. The bold numbers represent concordant calls. It can be seen that the major prediction errors are made between the two luminal types.

Table S3. Cell line properties

Cell Line	Origin	MaCSC Properties					Tumor Type	Ref
		K8+K14+	Self Renewal	Mammo-spheres	Alveolar Differentiation	Tumori-genesis		
Py230	Primary C57Bl/6 PyVmT tumor	Yes	Yes	Yes	Yes	Yes	Luminal	10
Py9813	Primary FVB/N PyVmT tumor	Yes	Yes	Yes	Yes	Yes	Luminal	
Py117	In vivo passage of Py230 tumor	Yes	Yes	Yes	Weak	Yes	Basal	
Py15-4	In vivo passage of Py230 tumor	Yes (weak)	Yes	Yes	No	Yes	Claudin-low	
Py16-1	In vivo injection of DMSO Tx Py230 cells	Yes (weak)	Yes	Yes	No	Yes	Claudin-low	

Table S4. 37 geneset

gene name
1810046J19Rik
1700052N19Rik
Agr3
Agr2
Ar
Abcc3
Actr3b
BC048546
Bspry
Bub1
Cdh1
Car12
Ccnb2
Cgn
Cldn4
Creb3l4
Epn3
Erbb2
Esr1
Dnajc12
Grb7
Gata3
Il6st
Fbp1
Fa2h
Foxa1
Foxc1
Hsd17b2
Mal2
Myo6
Myb
Mia1
Psmd3
Tcap
Tmem45b
Slc39a6
Xbp1
