# Supplementary material for: Multipotent luminal mammary cancer stem cells model tumor heterogeneity
Source: Breast Cancer Res. 2015 Oct 14;17:137. doi: 10.1186/s13058-015-0615-y (PMC4606989; doi:10.1186/s13058-015-0615-y)
Supplement: Additional file 2: Figure S1. — Clonal MaCSC differentiation. A. Clonal single cells from FVB/N cell line Py9813 are K8K14-positive. Left panel stained with secondary antibody alone. B. Early colonies contain only double-positive cells. Scale bar 20 μm. C. In two-dimensional cultures, K8 + K14+ Py9813 cells can self-renew or differentiate to form single-positive K8 or K14-positive cells. Scale bar 20 μm. D. Py9813 cells form hollow mammospheres that show the presence of double-positive cells and K8 single-positive luminal cells. Single-positive myoepithelial cells are less commonly observed. Scale bar 20 μm. E. The percentage of double-positive, single-positive and double-negative cells in immunofluorescence-stained two-dimensional cultures. Analysis of variance showed the groups were highly significantly different, P <0.0001 and by Holm-Sidak’s multiple comparisons test, there were significantly fewer K8+ and K14+ cells compared with K8 + K14+ cells, P <0.0001, and significantly fewer K14+ cells than K8+ cells, P <0.01 in both cell lines. No double-negative cells were observed in cultures of either cell type. Data are means ± SEM. F. Cultures of control or 300 nM retinoic acid-treated Py9813 cells stained with oil red O. Scale bar 20 μm. (PDF 900 kb) [file 13058_2015_615_MOESM2_ESM.pdf]

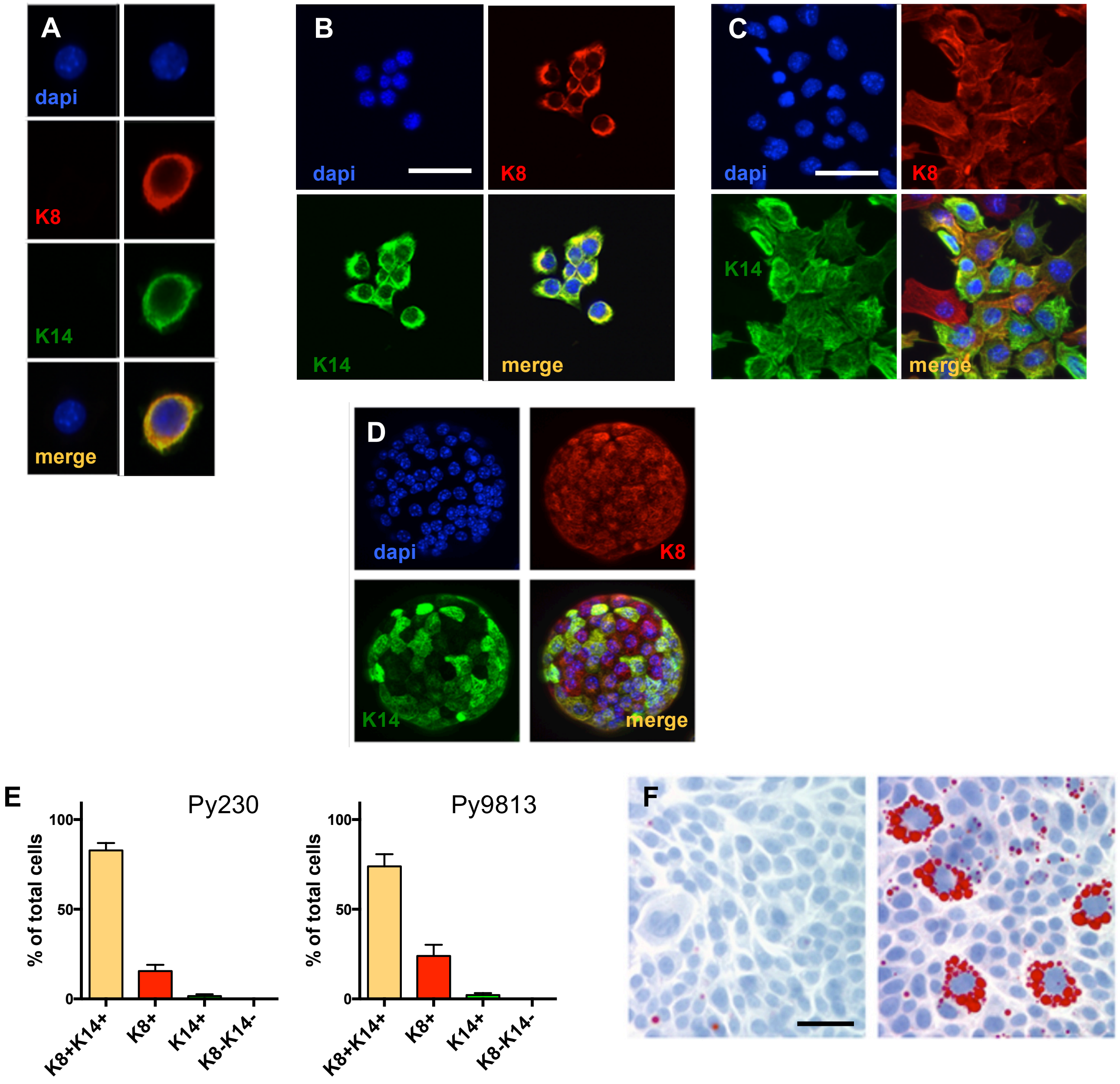

Figure S1. Clonal MaCSC differentiation. A. Clonal single cells from FVB/N cell line Py9813 are K8K14 positive. Left panel stained with secondary antibody alone. B. Early colonies contain only double positive cells. Scale bar 20 µm C. In 2-dimensional cultures, K8+K14+ Py9813 cells can self renew or differentiate to form single positive K8 or K14 positive cells. Scale bar 20 µm D. Py9813 cells form hollow mammospheres that show the presence of double positive cells and K8 single positive luminal cells. Single positive myoepithelial cells are less commonly observed. Scale bar 20 µm E. The percentage of double positive, single positive and double negative cells in IF stained 2-D cultures. ANOVA showed the groups were highly significantly different, $p < 0.0001$ and by Holm-Sidak's multiple comparisons test, there were significantly fewer K8+ and K14+ cells compared with K8+K14+ cells, $p<0.0001$ and significantly fewer K14+ cells than K8+ cells, $p<0.01$ in both cell lines. No double negative cells were observed in cultures of either cell type. Data are means ± SEM. F. Cultures of control or 300 nM retinoic acid treated Py9813 cells stained with oil red O. Scale bar 20 µm.
