# Supplementary material for: Multipotent luminal mammary cancer stem cells model tumor heterogeneity
Source: Breast Cancer Res. 2015 Oct 14;17:137. doi: 10.1186/s13058-015-0615-y (PMC4606989; doi:10.1186/s13058-015-0615-y)
Supplement: Additional file 3: Figure S2. — Py230 MaCSCs recapitulate spontaneous tumorigenesis and metastasis. A. Left panel - A Py230 MIN outgrowth in the cleared mammary fat pad. Scale bar = 2 mm. Right panel - Higher magnification showing K5-positive cells (brown) lining the hyperplastic ductal structures. Scale bar = 100 μm. B. Tumor formed from a single Py230 cell injected into the cleared mammary fat pad of a three-week-old mouse. At 29 weeks the tumor showed microcystic and solid areas of adenocarcinoma. Scale bar 200 μm Lung metastases indicated by the white arrows were observed in whole mounts of the lung. Inset shows H&E-stained lung metastasis. Scale bar 100 μm. C. Lung metastases (black arrows) visualized in whole mounts of a lung from a wild-type mouse with a 10-cell Py230 tumor. Inset shows H&E-stained lung metastasis. Scale bar 100 μm. D. Percentage of tumors formed by varying numbers of Py230 cells injected in 2 mg/ml matrigel into the cleared mammary fat pads of three to four-week-old female mice. (PDF 911 kb) [file 13058_2015_615_MOESM3_ESM.pdf]

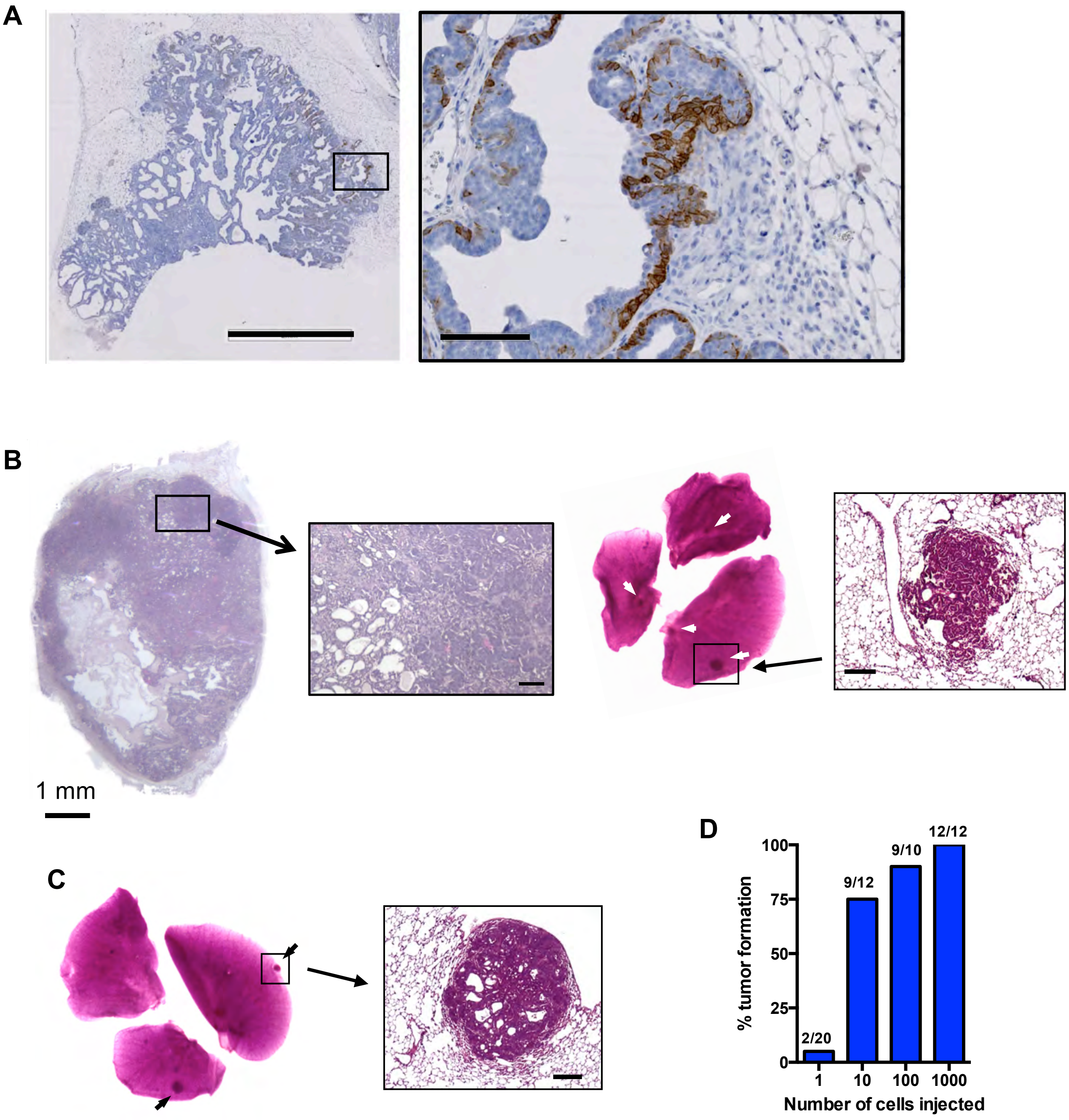

Figure S2. Py230 MaCSCs recapitulate spontaneous tumorigenesis and metastasis A. Left panel - A Py230 MIN outgrowth in the cleared mammary fat pad. Scale bar = 2 mm. Right panel – Higher magnification showing K5 positive cells (brown) lining the hyperplastic ductal structures. Scale bar = 100 µm. B.Tumor formed from a single Py230 cell injected into the cleared mammary fat pad of a 3-week old mouse. Inset - at 29 weeks the tumor showed microcystic and solid areas of adenocarcinoma. Scale bar 200 µm. Lung metastases indicated by the arrow white arrows were observed in whole mounts of the lung. Inset shows H&E stained lung metastasis. Scale bar 100 µm. C. Lung metastases (black arrows) visualized in whole mounts of a lung from a wild type mouse with a 10-cell Py230 tumor. Inset shows H&E stained lung metastasis. Scale bar 100 µm. D. Percentage of tumors formed by varying numbers of Py230 cells injected in 2 mg/ml matrigel into the cleared mammary fat pads of 3-4 week old female mice.
