# Supplementary material for: Multipotent luminal mammary cancer stem cells model tumor heterogeneity
Source: Breast Cancer Res. 2015 Oct 14;17:137. doi: 10.1186/s13058-015-0615-y (PMC4606989; doi:10.1186/s13058-015-0615-y)
Supplement: Additional file 4: Figure S3. — Py230 cells are estrogen responsive. A. Py230 cells cultured on glass coverslips for 48 hours in F12K/DMEM media with 5 % fetal calf serum. B. Py230 cells cultured on glass coverslips for 48 hours in F12K/DMEM media without phenol red with 5 % charcoal-treated serum. C, D. 104 × Py230 cells injected into intact or OVX female mice were monitored over an eight-week period. Tumor volume and final tumor burden data indicated that the majority of Py230 tumors were sensitive to the presence of endogenous estrogen. Data are means ± SEM from 16 tumors in four mice per group. E,F. Tumors from the same dataset that have undergone epigenetic and/or genetic changes resulting in increased growth (encircled in D) have been removed. Data are means ± SEM from 15 tumors in intact mice and 13 tumors in OVX mice. Data are representative of two similar experiments. (PDF 504 kb) [file 13058_2015_615_MOESM4_ESM.pdf]

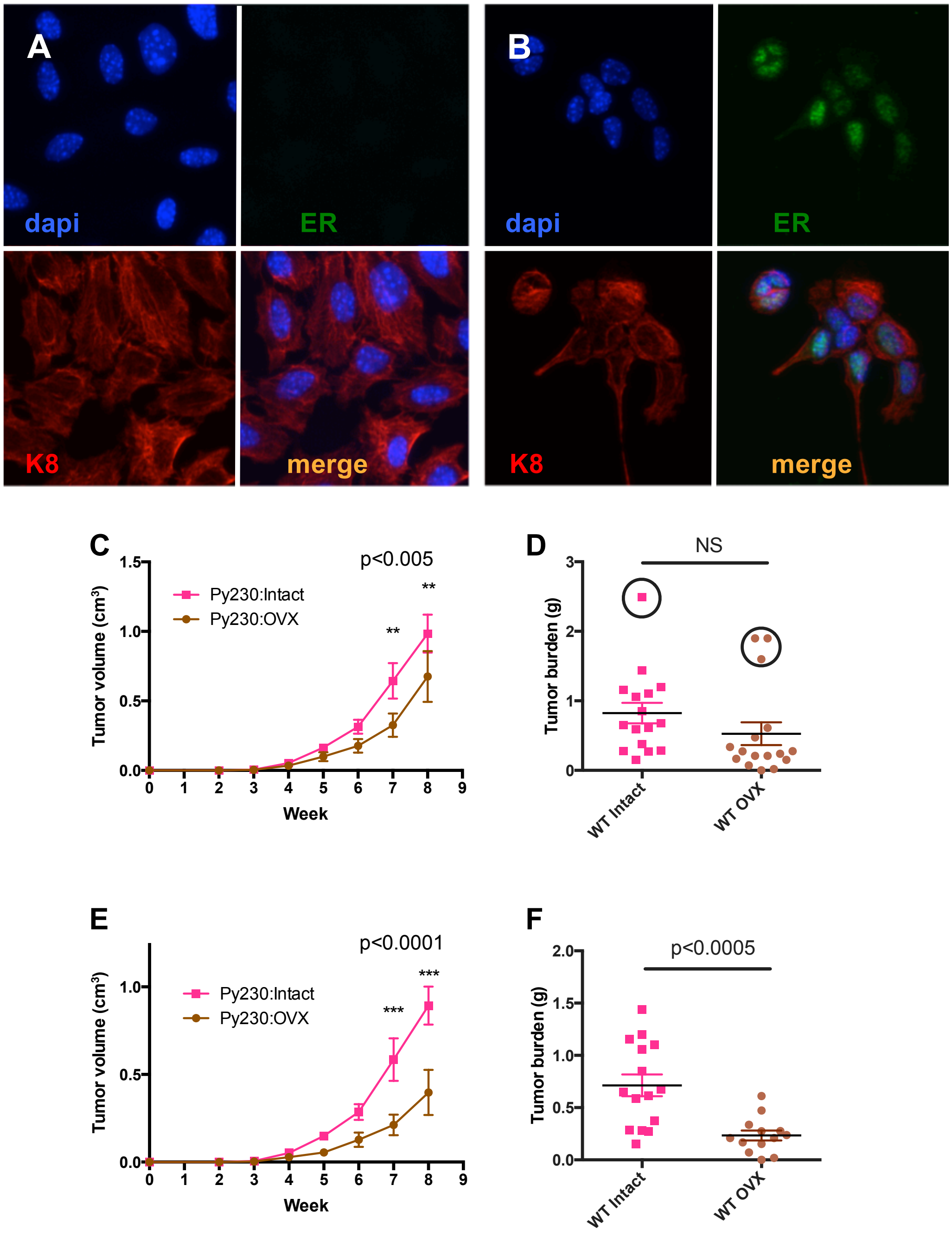

Figure S3. Py230 cells are estrogen responsive. A. Py230 cells cultured on glass coverslips for 48h in F12K/DMEM media with 5% fetal calf serum. B. Py230 cells cultured on glass coverslips for 48h in F12K/DMEM media without phenol red with 5% charcoal treated serum. C, D. 10^4 Py230 cells injected into intact or OVX female mice were monitored over an 8-week period. Tumor volume and final tumor burden data indicated that the majority of Py230 tumors were sensitive to the presence of endogenous estrogen. Data are means ± SEM from 16 tumors in 4 mice per group. E,F. Tumors from the same dataset that have undergone epigenetic/genetic changes resulting in increased growth (encircled in D) have been removed. Data are means ± SEM from 15 tumors in intact mice and 13 tumors in OVX mice. Data are representative of 2 similar experiments.
