# Supplementary material for: Multipotent luminal mammary cancer stem cells model tumor heterogeneity
Source: Breast Cancer Res. 2015 Oct 14;17:137. doi: 10.1186/s13058-015-0615-y (PMC4606989; doi:10.1186/s13058-015-0615-y)
Supplement: Additional file 5: Figure S4. — Validation of expression array data by GSEA and RT-PCR. A. Gene set for mammary luminal progenitor cells is significantly enriched (P <0.001) in luminal versus claudin-low tumors. B. Gene set for mammary stem cells is not enriched in luminal versus claudin-low tumors. C. Genes downregulated in mammary stem cells are significantly enriched (P <0.01) in the Py230 luminal cell line compared with the Py16-1 claudin-low cell line. D. Confirmation of lower expression of several mammary luminal progenitor-associated genes in Py16-1, a claudin-low cell line compared with the parental Py230 cell line. E. Confirmation of higher expression of several mammary stem cell-associated genes in Py16-1, a claudin-low cell line compared with the parental Py230 cell line. F. Higher expression of genes downregulated in mammary stem cells in luminal Py230 cell line compared with claudin-low Py16-1. For each gene comparison *P <0.05, ***P <0.005, ****P <0.001 with Sidak’s correction for multiple comparisons, following two-way analysis of variance. Data are means ± SEM for three independent cultures per group. G. 35 independent tumors were examined for expression of each of the indicated markers. The results were compared with expression by normal prelactating mammary gland (PLMG), which is the normal control for highly proliferative mammary tumors and spontaneous PyVmT tumors (n = 4 per group). (PDF 386 kb) [file 13058_2015_615_MOESM5_ESM.pdf]

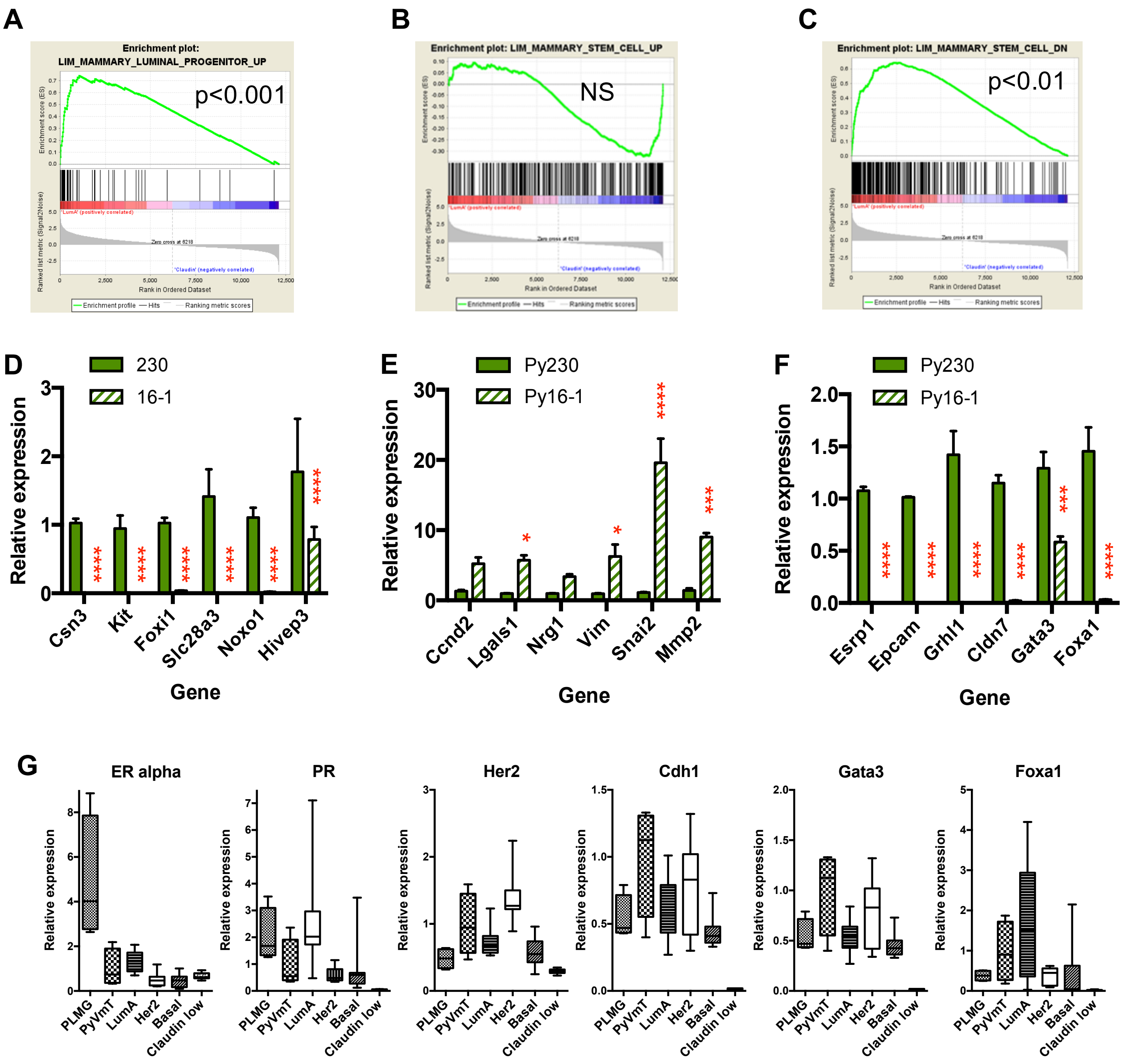

Figure S4. Validation of expression array data by GSEA and RT-PCR. A. Gene set for mammary luminal progenitor cells is significantly enriched ($p<0.001$) in luminal versus claudin-low tumors. B. Gene set for mammary stem cells is not enriched in luminal versus claudin-low tumors. C. Genes downregulated in mammary stem cells are significantly enriched ($p<0.01$) in the Py230 luminal cell line compared with the Py16-1 claudin-low cell line. D. Confirmation of lower expression of several mammary luminal progenitor associated genes in Py16-1, a claudin-low cell line compared with the parental Py230 cell line. E. Confirmation of higher expression of several mammary stem cell associated genes in Py16-1, a claudin-low cell line compared with the parental Py230 cell line. F. Higher expression of genes downregulated in mammary stem cells in luminal Py230 cell line compared with claudin-low Py16-1. For each gene comparison * $p<0.05$, *** $p<0.005$, **** $p<0.001$ with Sidak's correction for multiple comparisons following 2-way ANOVA. Data are means ± SEM for three independent cultures per group. G. 35 independent tumors were examined for expression of each of the indicated markers. The results were compared with expression by normal prelactating mammary gland (PLMG), which is the normal control for highly proliferative mammary tumors and spontaneous PyVmT tumors (n=4 per group).
