# Supplementary material for: Multipotent luminal mammary cancer stem cells model tumor heterogeneity
Source: Breast Cancer Res. 2015 Oct 14;17:137. doi: 10.1186/s13058-015-0615-y (PMC4606989; doi:10.1186/s13058-015-0615-y)
Supplement: Additional file 6: Figure S5. — Divergent mammary cancer stem cell phenotypes. A. Enrichment plot of p53 pathway genes in LumA or Claudin-low MaCSCs. B. Real-time PCR validation of the seven most highly differentially expressed genes in the p53 pathway analysis. Data are means ± SEM of six independent tumor samples per group. All groups were significantly different at P <0.005 or more using the Holm-Sidak correction for multiple comparisons. C. EMT-associated gene expression in Py230 and Py15-4 cell lines. D. Confluent cultures of Py230 and Py15-4 MaCSCs treated for 48 hours with dexamethasone and prolactin. Arrowheads indicate dome formation. E. Relative expression of ß casein in Py230 and Py15-4 cultures treated for 48 hours with dexamethasone and prolactin. (PDF 476 kb) [file 13058_2015_615_MOESM6_ESM.pdf]

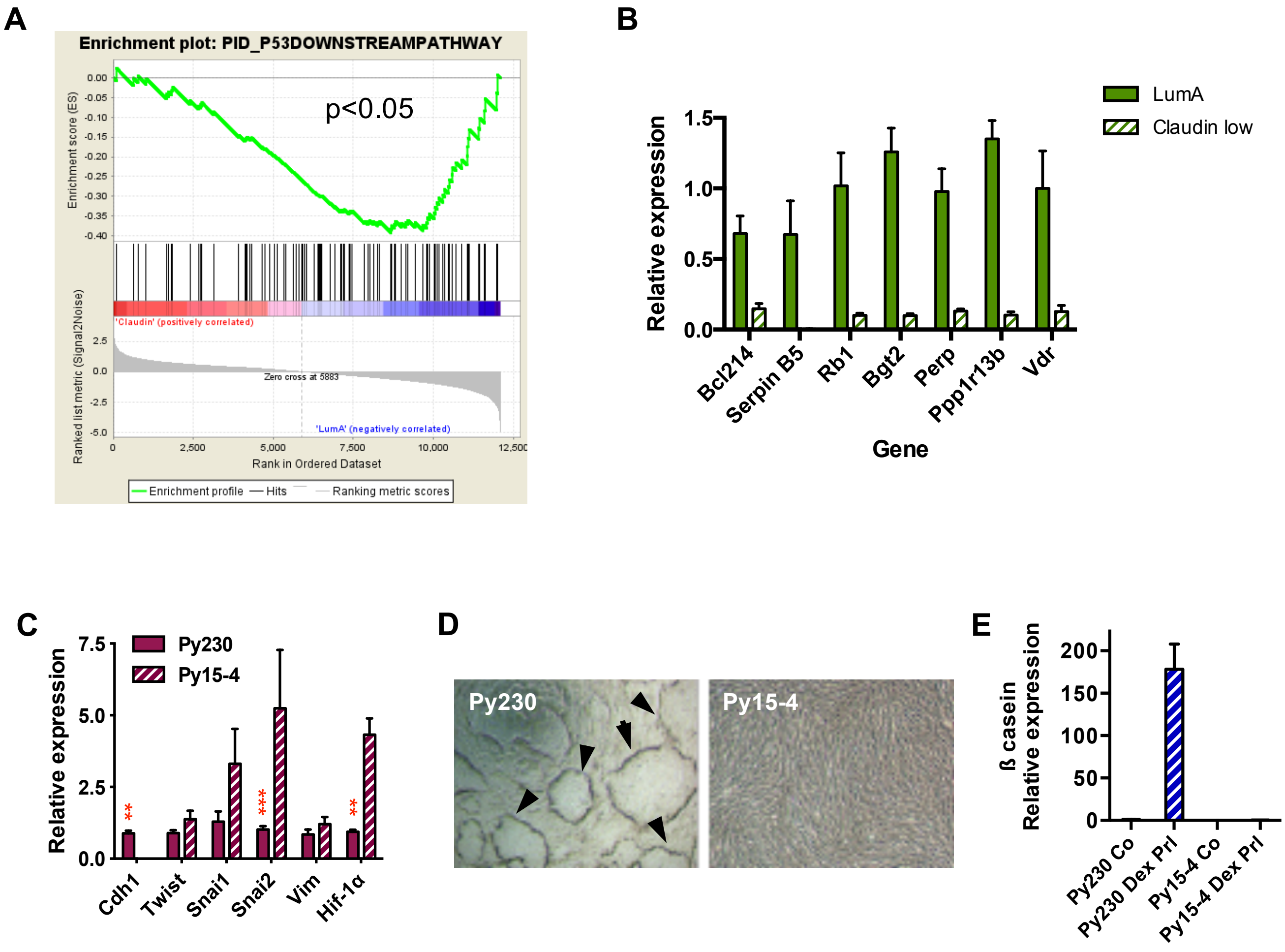

Figure S5. Divergent mammary cancer stem cell phenotypes. A. Enrichment plot of p53 pathway genes in LumA or Claudin-low MaCSCs. B. Real time PCR validation of the 7 most highly differentially expressed genes in the p53 pathway analysis. Data are means ± SEM of 6 independent tumor samples per group. All groups were significantly different at $p<0.005$ or more using the Holm-Sidak correction for multiple comparisons. C. EMT associated gene expression in Py230 and Py15-4 cell lines. D. Confluent cultures of Py230 and Py15-4 MaCSCs treated for 48h with dexamethasone and prolactin. Arrowheads indicate dome formation. E. Relative expression of ß casein in Py230 and Py15-4 cultures treated for 48h with dexamethasone and prolactin.
